# Forward-Looking Infrared Cameras for Micrometeorological Applications within Vineyards

**DOI:** 10.3390/s16091518

**Published:** 2016-09-18

**Authors:** Marwan Katurji, Peyman Zawar-Reza

**Affiliations:** Center for Atmopsheric Research, University of Canterbury, Christchurch 8140, New Zealand; peyman.zawar-reza@canterbury.ac.nz

**Keywords:** time sequential thermography, micrometeorology, self-organizing maps, surface energy balance, turbulence, microclimate, infrared camera

## Abstract

We apply the principles of atmospheric surface layer dynamics within a vineyard canopy to demonstrate the use of forward-looking infrared cameras measuring surface brightness temperature (spectrum bandwidth of 7.5 to 14 μm) at a relatively high temporal rate of 10 s. The temporal surface brightness signal over a few hours of the stable nighttime boundary layer, intermittently interrupted by periods of turbulent heat flux surges, was shown to be related to the observed meteorological measurements by an in situ eddy-covariance system, and reflected the above-canopy wind variability. The infrared raster images were collected and the resultant self-organized spatial cluster provided the meteorological context when compared to in situ data. The spatial brightness temperature pattern was explained in terms of the presence or absence of nighttime cloud cover and down-welling of long-wave radiation and the canopy turbulent heat flux. Time sequential thermography as demonstrated in this research provides positive evidence behind the application of thermal infrared cameras in the domain of micrometeorology, and to enhance our spatial understanding of turbulent eddy interactions with the surface.

## 1. Introduction

There is great interest in using near-target remote sensing techniques such as time-sequential thermography (TST) in precision agriculture, ecology [1] and phenomics [2]. Thermography techniques have to address the thermal condition of the object of interest and the thermal and humidity conditions of the intervening atmosphere. Near-surface atmospheric temperature is influenced by synoptic weather patterns and their interaction with local topography at the smaller scale, which together determines the nature of the air turbulence that envelops the plant and controls the rate of water vapor and heat exchanges. On the other hand, plants are more than passive objects and employ stomata to sense the surrounding environment and respond rapidly to abiotic stresses, such as the air temperature. Their response is typically through stomatal conductance to water vapor and/or transpiration, which are critical physiological controls. The plant’s surface temperature, or its brightness temperature as sensed by a thermal infrared camera, is the result of the interaction of the air temperature and the plant’s physiological response. Thus, to understand the plant’s microclimate through thermography (or the environment that embodies the plant to a few orders of magnitude in spatial scale relative to the plant’s volume), it is important to understand the brightness temperature signal (measured by a infrared camera) as a function of near-surface meteorological parameters controlling the energy exchanges happening across the plant-environment envelope.

Land surface temperatures are influenced by surface energy balance [3] especially when horizontal advection processes are negligible. Surface temperature varies as a consequence of partitioning of net-all wave radiation (Q*, or the balance between solar and infrared radiation input and output to the surface) into the subsurface conduction of heat (Q_G_) and changes in sensible (Q_H_) and latent heat exchange (Q_E_) with the overlying atmosphere. On short time scales (less than an hour), radiative input is relatively constant, unless clouds interfere or overlying plant canopy causes rapid changes (flickering) in solar radiation [4]. Higher frequency (seconds to minutes) surface temperature fluctuations are a response to the turbulent sensible and latent heat fluxes. Turbulence, caused by eddy motion, is expected to control temperature fluctuations on the same length and time scales as the atmospheric eddy motions.

The brightness temperatures of objects within the surface layer were not typically considered in the atmospheric community as a proxy for near-surface turbulence, but as infrared cameras become cheaper and are able to record data at high spatial and temporal resolutions, it is now feasible to study turbulence through the acquisition of brightness temperature. One of the earlier studies to investigate the coupling between coherent turbulent structures and surface temperature over an agricultural field (maize canopy) employed a directional infrared thermometer (sampling at 10 Hz) in identifying ramp structures in the surface temperature signal of the canopy with significant correlation with fluctuations in the air temperature above the canopy [5]. Coherent structures were identified as temperature ramps in the surface and air temperature time series, with the magnitude of surface temperature ramps being significantly smaller than the air temperature ramps. Surface temperature ramps are caused by turbulent eddies mixing warmer (or cooler) air with cool (warm) air from aloft. A similar study was conducted over grass [3] and also found direct relationships between surface brightness temperatures and independently measured surface-layer turbulence parameters.

Application of time-sequential thermography (TST) to calculate urban sensible heat fluxes (from a building) was first demonstrated by Hoyano et al. [6], and was further developed conceptually by Voogt [7] as a method for viewing the “footprint” of the coherent flow structures, and it was later emphasized by Christen et al. [8] that brightness temperature fluctuations are largely controlled by atmospheric turbulence while the level of fluctuation becomes modulated by surface properties, especially its thermal admittance. The application of TST to detect large temperature fluctuations in the unstable surface layer to understand the turbulence structure has shown great promise in field experiments [9], and was successful in deriving surface wind velocities over simple grass areas [10] via the principle of turbulent eddy interaction with surface brightness temperatures.

As forward-looking infrared cameras become more affordable, TST will become an attractive method to measure the energy and moisture exchanges between the surface and overlying atmosphere. This research utilizes spatial brightness temperature data from infrared cameras looking onto a vineyard canopy. The canopy is also instrumented with an eddy covariance system measuring in situ turbulent and radiation fluxes and near-ground thermistor-based temperature sensors. The brightness temperature fluctuations (sampled sequentially over a nighttime period at a high frequency) are then used to interpret the spatial variability of the turbulent nature of the site using a combination of in situ metrological measurements and a pattern recognition algorithm (or self-organizing maps, SOM) applied to the acquired brightness temperature data. The SOM approach allows for clustering self-similar images into groups that could then be analyzed according to their unique meteorological context. This research highlights the significance and relevance of the methodology in terms of relating the brightness temperature variability to atmospheric turbulence, which also highlights the local meteorology. This approach is not only limited to vineyard applications and could be applied and assessed over various other crop types or surfaces.

## 2. Methods

### 2.1. Study Site and Instrumentation

**Figure 1:**
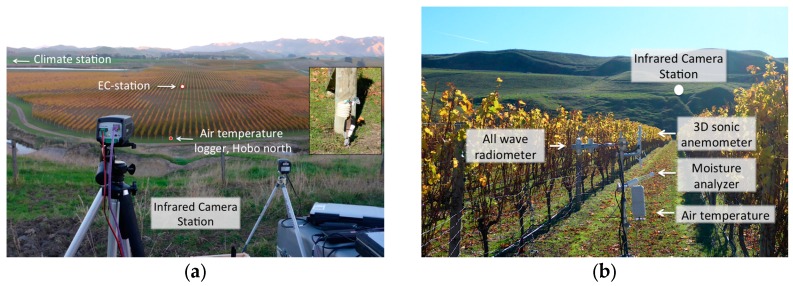
(**a**) The vineyard site covering most of the field of view of the two longwave infrared cameras shown on tripods in the foreground. The near-ground Hobo temperature logger is shown in the small figure inset; (**b**) A close up on the eddy covariance system (or EC-station in left panel) placed in the center of the camera’s field of view.

**Figure 2:**
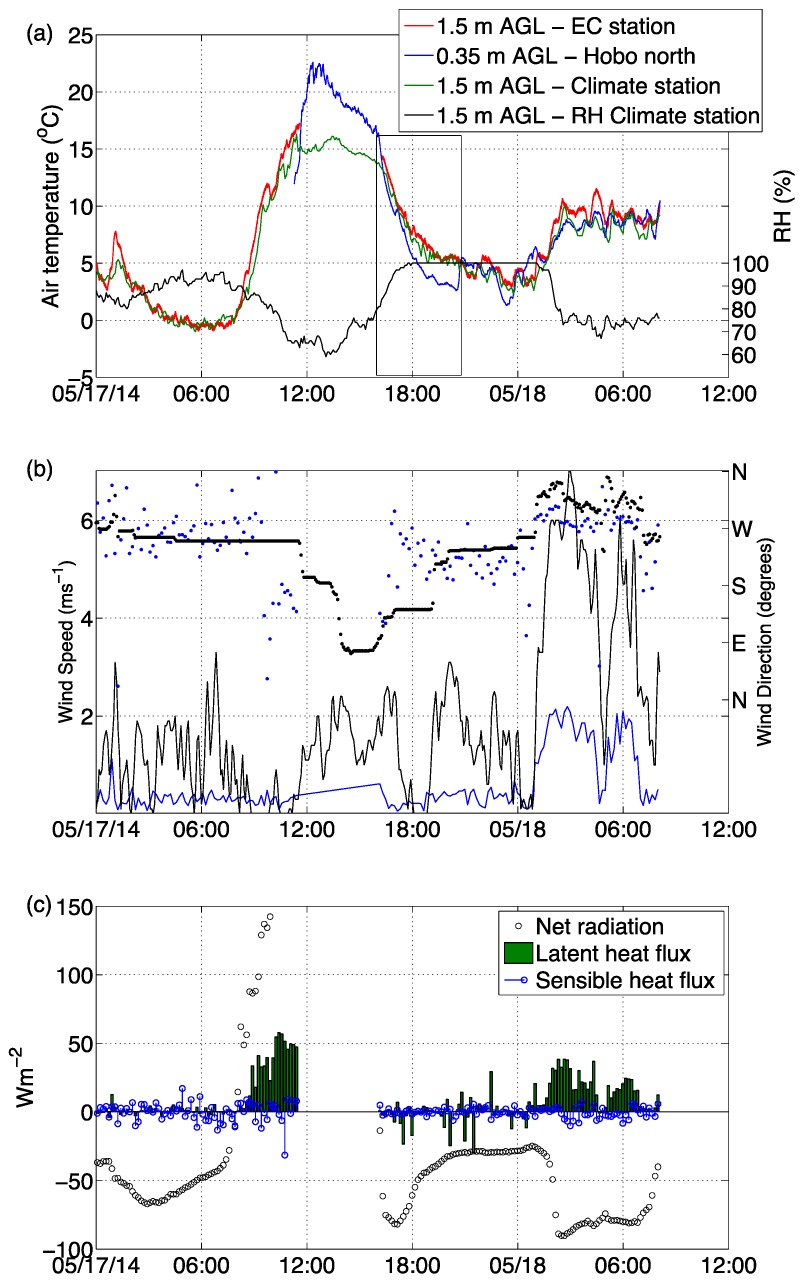
(**a**) Air temperature and relative humidity from within and above the vineyard canopy. The climate station data was collected from a low escarpment around 5 m above the canopy’s horizon. The black box shows the time period of the operation of the infrared cameras; (**b**) Wind speed (line) and direction (dotted) from within (blue) and above (black) the canopy; (**c**) The surface radiation and turbulent energy budget from the eddy covariance system inside the canopy.

The experimental site is located in Marlborough, situated in the South Island of New Zealand, renowned for abundance of vineyards and a major wine-producing region. The experimental site was chosen to be at the Lions Back vineyard in Seddon (41°41′51.5′′S, 174°05′13.6′′E) (Figure 1). This vineyard is ideal for the purpose of this experiment as it is easy to access and a nearby-elevated escarpment provides an ideal platform for placement of the infrared cameras overlooking most of the vineyard. Within the field of view of the cameras we have placed an eddy covariance system containing a 3D sonic anemometer, water vapor analyzer, measurements of all-wave radiation, and near surface air and soil temperature loggers in the same measurement area (Table 1). All measurements were controlled through data logging devices and/or monitored manually throughout the sampling period. The meteorological variables measured by the eddy covariance system (surface energy balance measurement system), climate station (standard meteorological parameters), and the near surface temperature logger (thermistor-based temperature sensing) were taken from 17 May 2014 12 a.m. up until 18 May 2014 9 p.m. While the thermography data was collected during the evening of the 17 May 2014 between 5 p.m. and 9 p.m. local standard time, which is also highlighted with a black rectangular box in Figure 2a.

### 2.2. Self-Organizing Maps, SOMs

The SOMs algorithm for the pattern recognition used in this analysis is SOM_PAK, found at Helsinki University of Technology website [11]. SOM iterates through the input dataset while matching each input to the SOM node that is closest in terms of its Euclidean distance, and then adjusts the node and its neighbors to incorporate the input data. A learning rate parameter controls the rate at which the SOM absorbs the information from the input data while a neighborhood radius determines which other nodes, are affected by the input data. The learning rate decreases to zero and the neighborhood radius decreases to one as the algorithm iterates through the dataset. Several matrix sizes (representing the number of maps or patterns) were tested, and for each matrix size the number of iterations and learning rate function types were adjusted, with the aim of reducing the quantization error, or the mean Euclidean distance between the input data and the SOM; bigger matrices generally exhibit lower errors. When the change in the error is minimal, the process can be considered complete. Criterion referred to as the “Sammon Map” is used to inspect whether each node of the SOM has more in common with its neighboring nodes, than non-neighboring. If not, the SOM algorithm was rerun with adjusted parameters. For examples on using SOM for various sources of meteorological data see [12,13,14,15,16,17,18].

The brightness temperatures (180 × 180 pixels) extracted as a spatial subset from the total infrared camera image (640 × 480 pixels) were used as input data for the SOM algorithm. First the pixel-wise data was normalized so that all pixel variables have a variance of 1, this allows for a more effective way in extracting patterns without biasing regions towards extreme values. Before executing the SOM algorithm the infrared brightness temperature perturbations (Tb’) were calculated based on the deviation of every sample (at 10 s interval) from the 10 min average. The de-trended Tb’ were then related to the turbulence and/or radiation forcing measured by the eddy covariance unit. The number of nodes (or pattern groups) was chosen to be a 3 × 3 matrix arrangement after testing with several other arrangements and an optimization between the size of the matrix and the detail of output was reached. After constructing the SOM, the non-clustered data was then matched with its most representative node (or spatial pattern), and then a number count was found to compare the relative population of each node with the original or non-clustered data. As a result, all 9 nodes had best matching units between 60 and 120, which suggests that the nodes were relatively well populated.

## 3. Results

### 3.1. Micrometeorological Context

The experiment extends between midnight of 17 May 2014 up until the early evening of 18 May 2014. During this period the region was synoptically quiescent, which limited surface wind speeds to less than 3 m·s^−1^, and the diurnal temperature ranged between just below freezing level and 20 °C (Figure 2). The air temperatures measured from within the vineyard canopy (EC station), near the surface air (Hobo north) and from a nearby climate station (Figure 2a) all show similar diurnal temperature variations within the experimental domain. The first morning period (17 May 12 a.m. to 8 a.m.) was colder by 5 to 10 °C than the following morning period (18 May 12 a.m. to 8 a.m.), mainly due to the more stable atmosphere maintained by weak surface wind speeds and the surface radiation cooling process. The following early morning wind speeds measured at 6 m above ground level (AGL) at the nearby climate station site increased up to 6 m·s^−1^, causing higher levels of turbulence within the canopy as registered by the increase in the sonic wind speed (Figure 2b) and the increase in the latent heat flux during the period between 12 a.m. and 8 a.m.) on 18 May. The relative humidity during this period (70%) was also lower than the night before (90%, Figure 2b), and the wind direction was from the northwest sector which blows relatively dryer air from the elevated mountainous regions.

### 3.2. Brightness and Air Temperature Relationship

**Figure 3:**
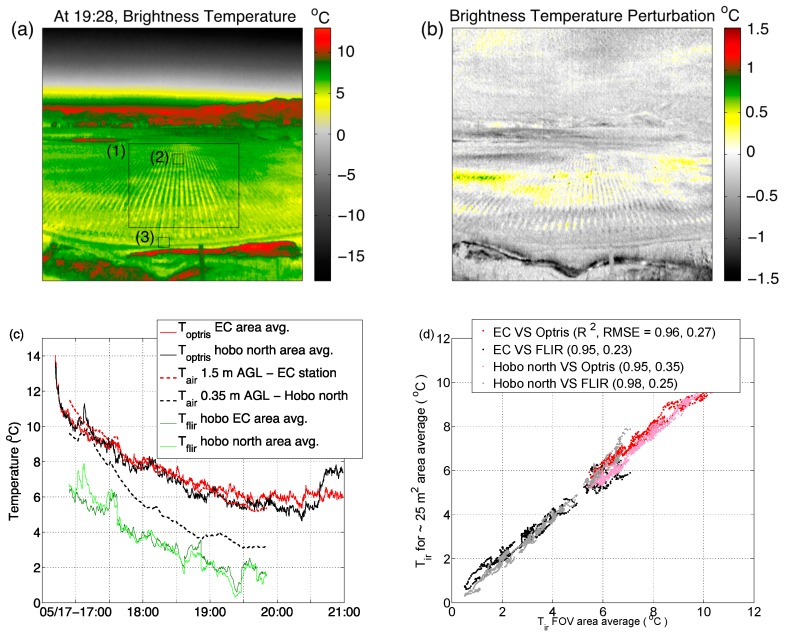
(**a**) A sample snapshot in time of the brightness temperature measured by the Optris camera. Regions (1), (2) and (3) represent the areas from which the mean was calculated from some of the analysis; (**b**) The same snapshot in time as in (**a**) but for the derived perturbation brightness temperature calculated from the deviation of each of the 10 s samples from the 10 min mean. Positive values show areas of increasing temperature in time; (**c**) Time series comparison of brightness temperature from two cameras, air temperature from the eddy covariance station and near-surface Hobo temperature logger; (**d**) Scatter plot of brightness temperature and in situ air temperatures.

In this section we aim to present a direct comparison between the measured brightness temperatures as seen by both of the long-wave infrared cameras and in situ air temperatures measured from within the canopy and from the surface at the northern edge of the canopy. The method we have used relies on averaging the brightness temperature over a 25 m^2^ area and a larger field of view area (see boxes (1), (2) and (3) in Figure 3a). Figure 3a shows a snapshot from the Optris camera of the entire field of view taken at 7:28 p.m. on 17 May 2014 local standard time. The color scale in Figure 3a represents the brightness temperature and a clear depiction of the warmer vegetated canopy (around 7 °C) rows running north to south and a cooler grass surface between the canopy rows with a brightness temperature of around 4 °C. Figure 3b is a derived image that represents the brightness temperature perturbation calculated by de-trending each sampled pixel from the temporal mean over a 10 min period. This statistical quantity represents a perturbation value that clearly shows pixels and regions that are either warmer (positive) or cooler (negative) than their neighbors. The resulting image highlights clouds in the upper sky section of the image that were invisible in Figure 3a, and warmer structures over the canopy. Figure 3c presents a time series of the area-averaged brightness temperature measured by the FLIR and Optris cameras over regions (2) and (3). A time series of the air temperature as measured by the EC-station and the HOBO temperature logger is also added to the figure to relate the brightness temperature to the air temperature as a function of the height of the air temperature measurement and location with respect to the vegetated canopy.

### 3.3. Brightness Temperature and Turbulent Heat Flux

**Figure 4:**
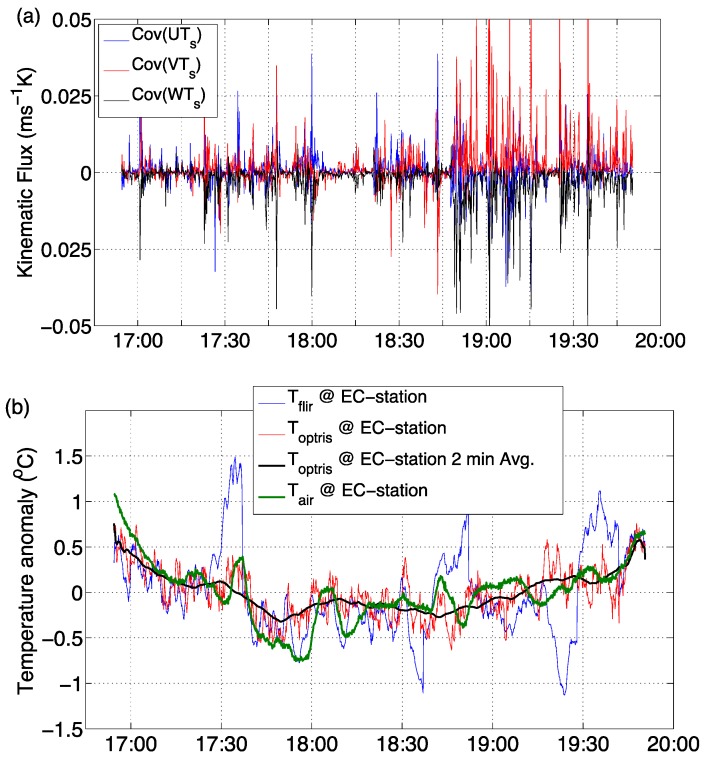
(**a**) Turbulent kinematic heat flux in the three Cartesian directions (U for east-west, V for north-south, and W in the vertical). Statistically, the heat flux was calculated based on the covariance of the velocity and sonic temperature; (**b**) Time series of brightness and air temperature at the eddy covariance area-averaged site.

The horizontal and vertical kinematic heat flux components were calculated from the covariance of the horizontal (U, V) and vertical (W) velocity components and the sonic temperature recorded by the sonic anemometer. In Figure 4a the kinematic heat flux is sampled at 10 s periods from collected data at 20 Hz; the results show turbulent horizontal and vertical heat advection over the few hours of the evening when the brightness temperature was sampled via the infrared cameras. Figure 4a shows an initial period of moderate to little turbulent heat flux (5 p.m. to 6:30 p.m.), with an increase of heat flux over the rest of the evening. This result is also supported by an increase in above-canopy wind speeds after 6 p.m. as depicted by the climate station wind speed data in Figure 2b. Figure 4b shows the area-averaged (area (2) in Figure 3a) de-trended brightness temperature from the FLIR and Optris cameras around the EC-station, and the corresponding de-trended air temperatures form the EC-station in the green line. 

### 3.4. Self-Organizing Maps (SOMs) of Brightness Temperature

**Figure 5:**
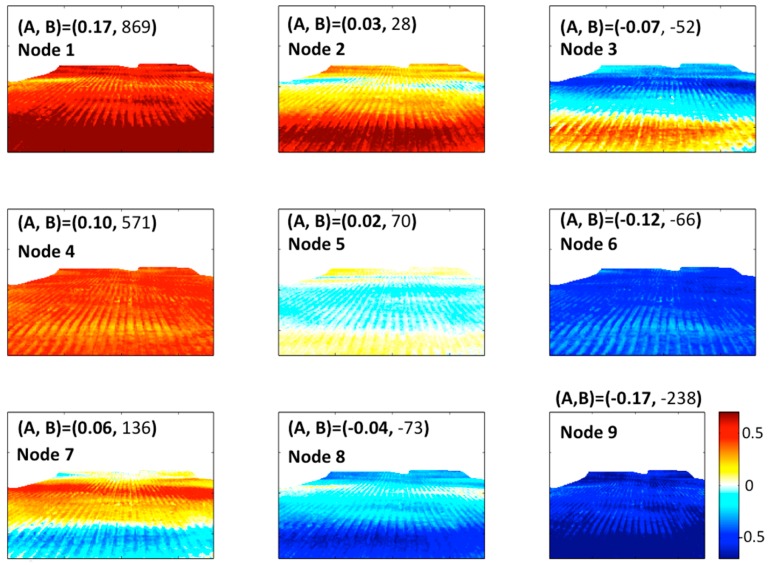
SOM nodes derived from the perturbation brightness temperature. Positive or red values or colors indicate an increase in Tir and negative or blue values or colors indicate a decrease in Tir or surface cooling. The numbers (A, B) on the top of each node are derived from the best matching units of that specific node and are perturbations of (A = average sky brightness perturbation temperature, B = average of the ratio of A by the absolute sum of the three kinematic turbulent heat flux components that were used in Figure 4a).

In this section we relate the spatial pattern of the brightness temperature to the meteorological conditions observed at the center of the image via the eddy covariance system. An unsupervised pattern recognition algorithm (SOM: see descriptions in the Methods section) was used to cluster the brightness temperature field into nine different nodes. This method allows us to interpret the brightness temperature field within the right meteorological context when other parameters (such as data from the eddy covariance system) are composited as a function of individual clusters. Figure 5 is the resulting SOM of all of the nearly 1000 images that were taken at a 10 s sampling interval from the Optris infrared camera; the sky brightness temperatures (appearing as a white mask at the top half of the nodes) were not used in the clustering but used as a derived composite variable for further analysis. The variables, which appear in parentheses in Figure 5 and are illustrated in Equations (1) and (2), represent the (A) mean sky brightness temperature perturbation, and the (B) ratio of the mean sky brightness temperature perturbation to the mean of the absolute sum of the three kinematic turbulence heat flux components derived earlier for Figure 4a.

(1) $$A\;\left(per\;node\right)=\frac{\sum_{i=1}^{n}\overset{=}{{Tb}^{\prime }\_sky}}{n}$$

where n is the number of images per node.

$\overset{=}{{\text{Tb}}^{\prime }\_\text{sky}}$ is the spatial average of the de-trended sky brightness temperature ${\text{Tb}}^{\prime }\_\text{sky}=\text{Tb}\_\text{sky}-\bar{\text{Tb\_sky}}$, where Tb_sky is the instantaneous pixel-based value and $\bar{\text{Tb\_sky}}$ is the 10 min average.

(2) $$B\;\left(per\;node\right)=\frac{A\;\left(per\;node\right)}{\left|cov\left(UTs\right)\right|+\left|cov\left(VTs\right)\right|+\left|cov\left(WTs\right)\right|}$$

## 4. Discussion

The results comparing the brightness temperature measured by the Optris infrared camera and in situ air temperatures show a very good match for the canopy height air temperature measurement and a warm bias for the air temperature measurement at the near-ground level (Figure 3c). The FLIR camera results show a systematic cold bias, with larger temperature oscillations when compared with the Optris, which could be explained by the automatic focusing method employed by the FLIR camera, which tends to periodically auto-sharpen the image, but also could be explained by the need for a camera calibration. Figure 3d shows a correlation diagram between the brightness temperature and the in situ air temperature measurement between the smaller area-averaged regions (1) and (2) and the larger field of view region (3). The results show a good linear correlation and a low value of root mean square error; they also show the cold bias offset previously revealed by the FLIR camera. This results also shows that the one-to-one relationship between the brightness temperature and the in situ air temperature is still preserved while spatially up-scaling the image over a homogenous terrain.

The brightness temperature signal of the Optris camera when compared to the direct measurements of turbulent heat flux (shown in red in Figure 4b) follows the air temperature trend and responds to the cooling and warming period suggested by the heat flux advection. The brightness temperature oscillation range also scales to the ranges shown by the air temperature record. The FLIR brightness temperature trend (shown in blue in Figure 4b) generally follows the initial cooling and then warming cycle but tends to overestimate the range with around five relatively large peaks. These peaks are linked to the automatic focusing of this specific infrared camera. Between 5 p.m. and 6:30 p.m. the brightness and air temperature trend did not exhibit either a positive or negative trend in comparison with the negative (cooling) or warming (positive) trends outside this period. This period also reflects a period of quiescence and little to no turbulent heat flux as shown in Figure 4a.

The unsupervised clustering carried out by the SOM technique in Figure 5 was successful in distinguishing nine clusters that have a meteorological context when compared to in situ measurements. The color in Figure 5 represents the brightness temperature perturbation (red being a warming trend and blue a cooling trend). The SOM shows distinct features that are relatively different among nodes. For example, node 1 shows a field-wide warming trend, while node 9 a field-wide cooling trend. Nodes 4 and 6 show the same extremes but with lower magnitudes, while nodes 3 and 7 show a north to south cooling or warming gradient and appear to represent an opposite brightness temperature gradient. In order to link the SOM patterns to a meteorological context we have composited two different variables (A and B in Figure 5 and Equations (1) and (2)) from the un-clustered data behind the construction of each of the node patterns. The mean sky brightness temperature perturbations, or A (varying between −0.17 and 0.17), correlate well with the brightness temperature trends. Positive values of quantity A (such as in nodes 1 and 4 for example) indicate a warming sky brightness temperature that relates to nocturnal cloud cover, which reradiates long-wave radiation back onto the surface, creating a homogenous spatial brightness temperature positive trend. The opposite applies for clear sky conditions (for example nodes 6 and 9). This result does not apply for nodes 2, 3, 5, 7, and 8 which show an order of magnitude lower mean sky brightness temperature perturbation and higher values of turbulent heat flux, which clearly creates localized and distinct warming and cooling trends within the brightness temperature spatial pattern. The particular patterns shown by nodes 3 and 7 are intriguing, as one could hypothesize that these opposite patterns are related to turbulent advection or mixing events that are happening within this local topographic catchment, especially as these patterns only exist during high turbulent heat flux periods (see quantity B for these nodes in comparison to quantity A for the other nodes).

## 5. Conclusions

We have demonstrated the use of forward-looking infrared cameras measuring the surface brightness temperature over a vineyard in the spectrum bandwidth of 7.5 to 14 μm at a relatively high temporal rate of 10 s for the application of vineyard-scale micrometeorology. Our results show that this technique, when applied for interpreting the micrometeorology as a function of cloud cover and within-canopy turbulence, could become a useful tool for up-scaling point measurements to spatially wide footprints. The temporal surface brightness signal over a few hours of the stable nighttime boundary layer intermittently interrupted by periods of turbulent heat advection was shown to be related to the atmospheric surface-layer dynamics observed by the eddy-covariance measurements, and reflects the temporal evolution of above-canopy wind variability. 

The analysis also introduced the SOM of the spatio-temporal brightness temperature data to reduce the dimensionality of this large dataset, but more importantly to highlight the physical dynamics of nighttime surface brightness temperature over a complex canopy measured by an infrared camera. The resultant spatial clusters were self-organized and compared to the meteorological context they reflected, and the spatial brightness temperature pattern was explained in terms of the presence or absence of nighttime cloud cover and down-welling of long-wave radiation and the canopy turbulence heat flux. Time sequential thermography as demonstrated in this research provides positive evidence behind the application of thermal infrared cameras in the domain of micrometeorology. The results of this experiment could then be used in accordance with the surface renewal theory (which assumes that surface-atmospheric turbulence exchanges are driven by ramp-like structures within the temperature time series), which will eventually allow for a spatial pixel-based derivation of sensible and latent heat flux which are essential for the canopy’s water balance during daytime periods [19,20,21,22].

There are a couple of limitations to this study that need to be considered when it is applied for more complex terrain. The first limitation comes from the potential effect of air temperature and humidity fluctuations along the camera’s line of sight on the interpretation of surface brightness fluctuations. This effect is usually addressed by simple one-dimensional radiative transfer modeling, which delineates the role of infrared signal attenuation. Both of these effects have been previously found to be less than 10% (for atmospheric temperature) and less than 3% (for atmospheric humidity) [8] for an urban setting and results may vary for other applications. The other limitation is the variable image pixel resolution as a function of depth. So the pixels furthest away from the camera have a different pixel resolution than pixels close to the camera. This could be only fixed with ortho-rectification when a high-resolution digital elevation map (DEM) for the site is available. A DEM was not available for this study, and given that the focus of this study was not to study atmospheric turbulence as a function of length scale, we considered that not affecting our major conclusions in this study.

## Figures and Tables

**Table 1:** Instrumentation specifications and measured variables.

Instrument	Description	Measured Variable	Range and Accuracy	Sampling Frequency
Campbell Scientific CSAT3	Three-dimensional ultrasonic anemometer	Cartesian components of velocity and sonic temperature (u, v, w, Ts)	±65 m·s^−1^ ± 0.08 m·s^−1^ for u, v ±0.04 m·s^−1^ for w, and −30 to 50 °C ± 0.01 °C for Ts	20 Hz
LICOR-7500	Open path infrared H_2_O analyzer (situated 30 cm below the sonic anemometer)	Specific humidity	0 to 60 parts per trillion (ppt) ±0.6 ppt	20 Hz
Kipp and Zonen CNR1	Net radiometer	Incident and reflected long- and short-wave radiation components	±10% over 24 h	1 Hz
Vaisala HMP45C	Temperature and relative humidity probe	Air temperature, soil surface temperature, and relative humidity	−40 to +60 °C ± 0.3 at 0 °C 0 to 90% ± 2%	1 Hz
HOBO U23	Radiation shielded temperature sensor	Air temperature at 35 cm above ground level or AGL	−40 to +70 °C ± 0.2 °C	0.1 Hz
FLIR A644sc	Uncooled infrared camera	Brightness temperature on a raster of 640 × 480 pixels	−40 to 150 °C ± 2 °C Spectral range 7.5 to 14 μm Thermal sensitivity 30 mK	50 Hz
Optris Pi 640	Uncooled infrared camera	Brightness temperature on raster of 640 × 480 pixels	−20 to 900 °C ± 2°C Spectral range 7.5 to 13 μm Thermal sensitivity 75 mK	0.1 Hz
